# Premotor and Posterior Parietal Cortex Activity is Increased for Slow, as well as Fast Walking Poststroke: An fNIRS Study

**DOI:** 10.1155/2023/2403175

**Published:** 2023-10-13

**Authors:** Shannon B. Lim, Sue Peters, Chieh-ling Yang, Lara A. Boyd, Teresa Liu-Ambrose, Janice J. Eng

**Affiliations:** ^1^Department of Physical Therapy, University of British Columbia, Vancouver, BC, Canada; ^2^Rehabilitation Research Program, GF Strong Rehabilitation Centre, Vancouver, BC, Canada; ^3^School of Physical Therapy, Western University, London, ON, Canada; ^4^Department of Occupational Therapy and Graduate Institute of Behavioral Sciences, College of Medicine, Chang Gung University, Taoyuan City, Taiwan; ^5^The David Mowafaghian Centre for Brain Health, University of British Columbia, Vancouver, BC, Canada; ^6^Centre for Aging SMART at Vancouver Coastal Health, Vancouver, BC, Canada

## Abstract

**Methods:**

Twenty individuals in the chronic stage of stroke walked: (1) at their normal pace, (2) slower than normal, and (3) as fast as possible. Functional near-infrared spectroscopy was used to assess bilateral prefrontal, premotor, sensorimotor, and posterior parietal cortices during walking.

**Results:**

No significant differences in laterality were observed between walking speeds. The ipsilesional prefrontal cortex was overall more active than the contralesional prefrontal cortex. Premotor and posterior parietal cortex activity were larger during slow and fast walking compared to normal-paced walking with no differences between slow and fast walking. Greater increases in brain activation in the ipsilesional prefrontal cortex during fast compared to normal-paced walking related to greater gait speed modulation.

**Conclusions:**

Brain activation is not linearly related to gait speed. Ipsilesional prefrontal cortex, bilateral premotor, and bilateral posterior parietal cortices are important areas for gait speed modulation and could be an area of interest for neurostimulation.

## 1. Introduction

While adequate gait speed is an important factor for successful ambulation within the community, the ability to change gait speed is also important for safe interaction with the environment. For example, the ability to appropriately increase gait speed is important for crossing the street before a light turns red and the ability to slow down gait is important before stepping up onto a curb. This ability to change walking speed has been related to balance performance [1] and falls risk [2]. In fact, older adults who demonstrate a decreased ability to adapt their walking speed are almost five times more likely to be at high risk of falls [3]. After a stroke, the ability to change gait speed is often impaired [4] and individuals who are not able to increase gait speed exhibit lower functional ambulation [5].

Investigation of brain activation while walking at different speeds may provide some insight into gait speed impairments. In healthy adults, slow walking (0.4–0.6 m/s) primarily results in activation of the premotor and supplementary motor area of both cortices [6]. As walking speed increases to 0.7 and 0.8 m/s, bilateral prefrontal and sensorimotor cortices also become active [6]. When healthy older adults were asked to walk at different speeds, the greatest activation was found at the fastest walking speed [7]. Harada et al. [7] also found that the prefrontal cortex activity increase was greater for individuals that have a slower fast gait speed (<1.67 m/s) compared to those who had a faster gait. In addition, when gait speed continuously changes on one leg using a split-belt treadmill, healthy adults show increased activation within the supplementary motor area and posterior parietal cortex compared to walking at a stable speed [8]. It is possible that impaired gait speed modulation poststroke may be explained by abnormal activation in these prefrontal, premotor, supplementary motor, sensorimotor, or posterior parietal cortical areas.

To date, the impact of stroke on functional activation changes with speed modulation is unknown. Previous studies have shown indications of asymmetric brain activations with greater ipsilesional prefrontal [9], contralesional sensorimotor [9, 10], and contralesional posterior parietal [9] cortices relating to faster walking speeds poststroke. The changes in the amplitude of activation during walking at different speeds poststroke are also unclear. Bansal et al. [11] recently demonstrated that their lower-functioning stroke group had a limited capacity to increase their gait speed compared to their higher-functioning stroke group. Although not measured in their study, it is possible that this impairment is related to their participant's capacity to activate certain brain regions.

To help understand the impact stroke has on gait speed modulation, the purposes of this study are to explore: (1) the symmetry of brain activation as individuals increase or decrease their gait speed, (2) the activation levels in frontal to parietal brain regions during walking at different speeds, and (3) the relationship between an individual's stroke impairment or their ability to modulate their gait speed and change in their brain activation. Specifically, we hypothesized that: (1) fast walking would show asymmetrical activity with greater ipsilesional prefrontal, contralesional sensorimotor, and contralesional posterior parietal activation; (2) a graded activation would be observed with a lower magnitude of activation observed in slow walking and the highest activation with fast walking; and (3) the magnitude of brain activation changes from fast and slow compared to normal-paced walking would relate to impairment and gait speed modulation ability.

## 2. Methods

### 2.1. Participant Recruitment

Participants were recruited through convenience sampling via posters at private clinics, local rehabilitation centers, and online platforms. Information regarding the study was also distributed through phone or mail to previous participants who have agreed to be contacted for future studies. Study details were approved by the University of British Columbia Research Ethics Board (H18-01003) and written and informed consent was provided by all participants.

### 2.2. Participant Screening

Individuals were screened for eligibility by telephone. Inclusion criteria were as follows: age greater or equal to 18 years; telephone minimental state exam greater than 21/26 [12, 13] indicating no moderate or severe cognitive impairment; stroke incident greater than 6 months previous (chronic stroke); single known stroke; one-sided hemiparesis; able to walk independently (gait aids allowed) for 1-min bouts; and able to understand and follow directions in English. Exclusion criteria were musculoskeletal injury impairing walking and neurological injury other than stroke.

### 2.3. Demographic Data

Age, sex, global cognition (using the Montreal Cognitive Assessment), and gait aid used were collected. Stroke details were obtained from medical charts when available. When medical charts were not available, details on time poststroke and stroke type (ischemic/hemorrhagic) were collected through verbal reports by the participants. Lesion location was determined from structural MRI obtained through medical records or collected for this study when eligible.

### 2.4. Task Procedure

The walking tasks were completed in a 50-m hallway. Participants completed one to two familiarization trials of each walking condition. Participants stood at either end of the hallway to begin each trial. The starting end was randomly determined by the researcher. After a minimum 30 s of quiet stance, a verbal “go” from the researcher specified the start of the walking trial; a verbal “stop” indicated the end of the trial. All participants were told to keep their head position consistent and to avoid unnecessary talking throughout the walking trials. Walking trials were 30 s long and were performed four to five times. A wheelchair and spotter were positioned behind the participant for safety during all trials. At the end of each walking trial, participants stood for 5 s before sitting in the wheelchair. The spotter then pushed the participant to the end of the hallway to start the next trial. All trials had at least 30 s of standing immediately before the start of the trial—this allowed for the brain signals to return to baseline.

Participants performed three walking conditions: normal-paced walking speed (*NORM*), slow speed (*SLOW*), and fast speed (*FAST*). Overground walking was chosen to resemble daily walking conditions and to investigate limitations to changing one's gait speed. All participants first completed the *NORM* condition and then either the *SLOW* or *FAST* condition (randomized). For the *SLOW* condition, participants were told to walk slower than their normal pace and for the *FAST* condition, participants were told to walk as fast as they could without running. The PychoPy3.0 program was used for randomizing the conditions and triggering/timing the trials [14].

### 2.5. Functional Brain Activation

Functional brain activity was measured by functional near-infrared spectroscopy (fNIRS). All data collection and analysis regarding fNIRS were completed in accordance with best practice guidelines [15, 16]. fNIRS was chosen, opposed to other imaging devices, due to its ability to be wireless, portable, and robust to motion artifacts. Further information on the advantages and details of fNIRS have been discussed in previous reviews [17–19]. The NIRSport2 (NIRx Medical Technology, Germany) was used, which had 16 LED emitters that released near-infrared light at 760 and 850 nm for measurement of both deoxygenated (HbR) and oxygenated (HbO) hemoglobin, respectively, and 23 silicon photodetectors. Optodes were connected to the fNIRS collection device, which was worn as a backpack by the participants. fNIRS data were continuous sampled at 4.36 Hz through Aurora 1.4 (NIRx Medical Technologies, Berlin, Germany). The probe configuration for this experiment was similar to our previous studies [9, 20, 21] with 48 long separation channels (∼30–35 mm apart) and 8 short separation channels (8 mm apart). Two different distances were chosen to control for extracerebral systemic changes such as breathing, heartbeat, and mayer waves [22]. As previous studies have primarily shown significant findings with HbO and little change in HbR, results will focus on HbO findings. HbO is also more reproducible and stable over time [23], has the highest correlation to fMRI BOLD measures [24]. For transparency, detailed HbR findings are reported in Tables S4 and S5.

### 2.6. Probabilistic Localization

Several approaches were taken to improve the accuracy of localizing functional brain activation. First, spatial locations of the optodes on each participant's scalp were digitally collected using a 3D digitizer (Polhemus Patriot, USA) and the software PHOEBE [25]. To control for the location of digitization within the optode holder (7 mm diameter), a custom interface between the optode holder and the digitizing stylus was 3D printed to consistently place the stylus at the center of the optode holder. The 3D digitations were then imported to AtlasViewer [26], which was used to project the channels to the Colin27 atlas brain. Montreal Neurological Institute coordinates and Automated Anatomical Labeling projections were provided by AtlasViewer and were then translated into Brodmann labels using the Allen Human Brain Atlas [27] and the Yale BioImage Suite Package web application [28]. This labeling system was then used to categorize channels into regions of interest: PFC, PMC (which was also combined with SMA), SMC, and PPC on the ipsilesional and contralesional hemispheres. Individual MRIs were then used to determine the location of the stroke lesion. If stroke lesions were present along the cortex, channels that were projected to lesion sites were removed (i.e., not analyzed).

### 2.7. Stroke Lower Extremity Impairment

The lower extremity portion of the Fugl-Meyer assessment [29] was used to determine motor impairment after stroke. This assessment was completed by a trained physiotherapist, has excellent inter- [30] and intra-rater [31] reliability, and is a recommended outcome measure for individuals living after stroke [32].

### 2.8. Gait Speed Modulation

Gait speeds were first calculated for every trial by determining the distance walked during the 30-s trials. Average gait speed was calculated for each condition and the differences in gait speed were calculated between *NORM* to *FAST* and between *NORM* to *SLOW*. These differences were used to determine gait speed modulation ability.

### 2.9. Analysis

To determine if the tasks were performed as intended, task performance differences in gait speed were determined by paired *t*-tests between NORM and FAST and between NORM and SLOW. A Bonferroni corrected alpha of 0.025 was used to reduce Type 1 error. HomER2 [33] was used to preprocess the fNIRS data. HomER2 functions and corresponding parameters are indicated within square brackets. First, noisy channels were removed (enPruneChannels: SNRtresh = 6.67, dRange = 5e-4 to 1e+00, SDrange:0−45) before converting the signal into optical density (hmrIntensity2OD). 0.5 s time windows were used to identify motion artifacts in data for signals exceeded either 20 standard deviations above the mean signal for each channel or a change greater than five times in amplitude (hmrMotionArtifactByChannel: tMotion = 0.5, tMask = 1.0, STDEVthresh = 20.0, AMPthresh = 5.00). Once identified, motion correction was applied using a wavelet transformation with a 1.5 interquartile range (hmrMotionCorrectWavelet: iqr = 1.5) [34–36]. The remaining motion artifacts were assessed again using the same parameters as above (hmrMotionArtifactByChannel: tMotion = 0.5, tMask = 1.0, STDEVthresh = 20.0, AMPthresh = 5.00). The number of channels removed from further analysis for each participant can be found in Tables S2 and S3. A lowpass filter of 0.15 Hz was then applied to the data (hmrBandpassFilt: lpf = 0.15) and converted to hemoglobin concentration using the modified Beer–Lambert equation (hmrOD2Conc: ppf = 6.0, 6.0) [37, 38]. A general linear model with an ordinary least squares approach [39, 40] and a 0.5 s width and 0.5 s step consecutive Gaussian basis function [41] was used to estimate the hemodynamic response. Superficial contributions to the signal were also removed by regressing out the data from the short separation channel that has the highest correlation to each channel [40–44]. Any drift within the signal was corrected using a third-order polynomial correction [40] (hmrDeconvHRF_DriftSS:trange = −20.0 35.0, glmSolveMethod = 1, idxBasis = 1, paramsBasis = 0.5 0.5, rhoSD_ssThresh = 15.0, flagSSmethod = 1, driftOrder = 3, flagMotionCorrect = 0). Once preprocessed, data were exported to a custom Matlab script for baseline corrections (−15 to 0 s before walking onset) and region of interest averaging. Brain activations during the task were calculated by averaging hemoglobin amplitudes during the first 20 s of walking. The first 20 s of the task were chosen for analysis, opposed to the entire 30-s walking task because some participants walked fast enough to reach the end of the straight walking track before the 30 s was up. Thus, 20 s was chosen as all participants were walking along the straight path during this period.

*Aim 1:* To assess activation symmetry, a laterality index was calculated for each region of interest [45]. The laterality index was calculated as follows:

(1) $$\begin{array}{c} \text{Laterality index}=\frac{\text{Ipsilesional activation}-\text{Contralesional activation}}{\left|\text{Ipsilesional activation}\right|+\left|\text{Contralesional activation}\right|}. \end{array}$$

Using this equation, a positive value indicates more activation in the ipsilesional hemisphere whereas a negative value indicates more activation in the contralesional hemisphere. The laterality index was then compared between conditions using linear mixed-effects models in the statistical package “lme4” within the R Studio software. Participants were set as random effects and condition (*NORM*, *FAST*, *SLOW*) were included as fixed effects.

*Aim 2:* To look at the effects of condition (*NORM*, *SLOW*, *FAST*) on brain activation, linear mixed-effects models (one for each region of interest: PFC, PMC, SMC, PPC) were used. Within this model, participants were included as random effects, condition was included as fixed effect, and if it significantly improved the model, hemisphere was also added as a fixed effect.

*Aim 3:* Relationships between changes in brain activation and stroke lower extremity impairment and gait speed modulation were determined using Pearson's correlations. Change in brain activation was calculated for each region of interest (PFC, PMC, SMC, PPC). These changes in brain activation were determined by calculating the difference between average brain activation during *NORM* and average brain activation during *FAST* or *SLOW. C*orrelations were assessed for each region separately: ipsilesional PFC, PMC, SMC, PPC and contralesional PFC, PMC, SMC, PPC.

All relevant assumptions and diagnostics were checked for each statistical test. Appropriate modifications were made and reported when necessary. Due to the relatively small sample size, results are reported using a standard alpha of 0.05 in order not to miss potential effects. Data are also shown with a Bonferroni correction for reducing Type 1 error by running multiple models (*p* ≤ 0.0125=0.05/4 models and regions) and a Benjamini–Hochberg false discovery rate of 5% to correct for multiple Pearson's correlations.

## 3. Results

Twenty-two individuals were eligible and consented to the study and 20 participants completed the study. Two eligible participants were not able to attend data collection sessions due to restrictions as a result of the COVID-19 pandemic. Average demographic data and gait performance are displayed in Table 1. Overall, participants completed the tasks as intended. Compared to the *NORM* condition, an average increase in gait speed of 30% was calculated for the *FAST* condition and an average decrease of 21% was calculated for the *SLOW* condition. One participant ended up increasing their gait speed during *SLOW* compared to *NORM* by 0.03 m/s, another participant was not able to increase their gait speed during the *FAST* condition compared to *NORM*. Further details on individual stroke demographics and performance can be found in Table S1.

**Figure 1:**
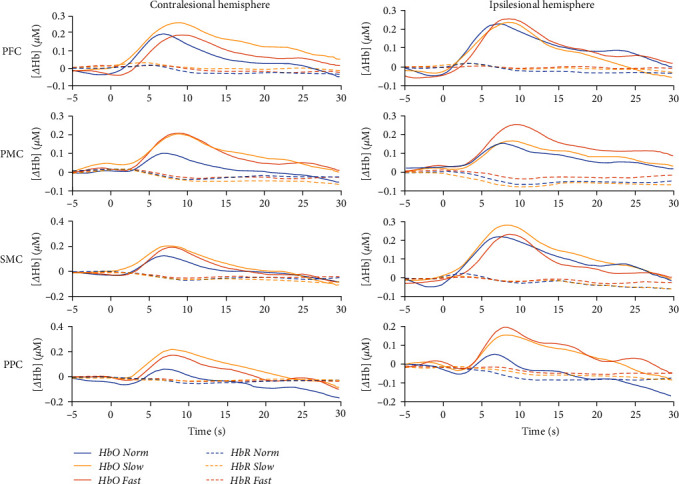
Average (*N* = 20) activation for oxyhemoglobin (HbO) and deoxyhemoglobin (HbR) during each walking condition. PFC = prefrontal cortex; PMC = premotor cortex; SMC = sensorimotor cortex; PPC = posterior parietal cortex.

The overall hemodynamic waveform showed a typical shape with increases in HbO and smaller amplitude decreases in HbR starting within the first 5 s of walking for all conditions (Figure 1).

**Figure 2:**
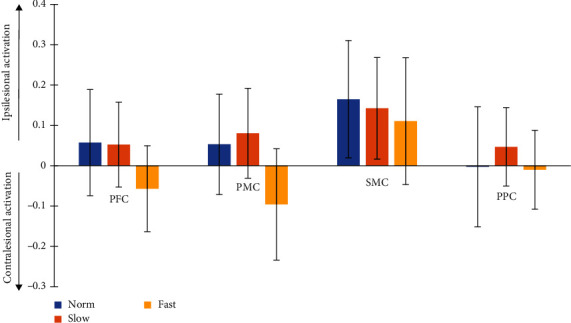
Average and standard error (*N* = 20) change in laterality index for HbO during each walking condition. Positive values indicate more activation along the ipsilesional hemisphere and negative values indicate more activation along the contralesional hemisphere. PFC = prefrontal cortex; PMC = premotor cortex; SMC = sensorimotor cortex; PPC = posterior parietal cortex.

 
*Aim 1:* No significant differences in laterality were observed between the three walking speeds (Figure 2 and Table 2, Aim 1).
 
*Aim 2:* Linear mixed models showed an effect of speed for the PMC and PPC regions. Compared to the *NORM* condition, *FAST* and *SLOW* conditions showed increased activation for both PMC and PPC. No significant differences were found between the *FAST* and *SLOW* conditions. Ipsilesional PFC showed significantly greater activation compared to the contralesional side (Table 2, Aim 2).
 
*Aim 3:* The magnitude of gait speed increase from *NORM* to *FAST* showed a moderate positive correlation to the amount of brain activation increase in ipsilesional PFC. Less impairment (i.e., higher FMLE scores) related to greater ipsilesional PFC activation changes from *NORM* to *FAST*, with a moderate but not significant correlation after correction for multiple comparisons. No significant relationships were found with the contralesional hemisphere (Table 3).

## 4. Discussion

This is the first study to investigate brain activation during modulation of gait speed poststroke. Results for Aim 1 were supportive of the null hypothesis while results for Aim 2 and 3 partially supported the alternate hypotheses.

### 4.1. Laterality

Although previous works have suggested some hemispheric differences with different walking speeds, the current study showed no changes in laterality with the three walking speeds. Despite not observing an overall change in hemispheric activation, participants exhibited a greater level of ipsilesional prefrontal cortex activation throughout. This is similar to our previous study where ipsilesional PFC was also elevated compared to the contralesional side and had a significant relationship with performance [8].

### 4.2. Premotor and Posterior Parietal Regions Involved in Speed Modulation

The current study showed that PMC and PPC play a role in modulating gait speeds, with the increase in PPC activity during *FAST* compared to *NORM* walking showing the largest difference. This increase in brain activity during *FAST* and *SLOW* walking occurred despite having participants of varying impairment levels (Fugl-Meyer lower extremity score range: 18–34) and gait speed abilities (normal-paced gait speed range: 0.14–1.39 m/s). Notably, no significant correlations were observed between PMC or PPC changes and impairment. This may indicate that these regions are involved in the task of changing gait speeds itself, and not as a regulator of the amount of gait speed change. Interestingly, our previous work in individuals poststroke showed that PMC activation does not change during normal-paced walking compared to standing [9]. PMC is known to be involved in motor planning and preparation [46, 47] and the supplementary motor area, which we included in our PMC region, is involved in online motor adjustments [8]. This finding suggests that continuous planning or movement preparation is not required for walking at a comfortable speed, which may arguably be quite automatic for the independent walkers in our studies, whereas a change from normal-paced walking requires more planning and online adjustments. The impact of a stroke on motor control and balance [48] may require these individuals to similarly activate PMC and PPC during both slow and fast walking—speeds they do not regularly walk at and, therefore, are more novel. Previous work has also suggested that PPC is important for gait adaptation [49, 50]. Both studies used a split-belt treadmill with healthy adults and showed involvement of the PPC by recording activation with electroencephalography [50] or suppressing the region with transcranial direct stimulation [49].

### 4.3. Ipsilesional PFC Activation Relates to Gait Speed Modulation and Impairment

The only significant correlation observed was with brain activation changes from *NORM* to *FAST* walking in ipsilesional PFC. Ipsilesional PFC activity was also overall higher compared to the contralesional side. PFC activity is often elevated when learning or performing a new task [51] or a complex task [52]. The complexity of walking at a different speed may require an increase in PFC whereby individuals who are able to perform the task better (i.e., greater gait speed modulation) and are less impaired (i.e., higher Fugl-Meyer scores) are able to increase PFC activation to a greater extent. PFC also plays an important role in regulating information relayed to various areas of the cortex, such as sensory information to the PPC [53]. Interestingly, we also showed the greatest PPC activation for *FAST* walking. Sauvage et al. [54], found increased PPC activation with their slow compared to fast leg movements and suggested that this was a result of a greater need for fine voluntary control requiring greater attention for selecting relevant sensory feedback. After stroke, sensory integration is often impaired and varies depending on the type of sensory information [53]. Impaired sensory integration may be observable through heightened PPC, indicating decreased overall efficiency, and an increased need for PFC to regulate the incoming sensory information for successful task performance.

Clinically, the results from our study indicate that PMC, PPC, and ipsilesional PFC may be important targets for noninvasive stimulation techniques such as repetitive transcranial magnetic stimulation or transcranial direct stimulation. Using these stimulation techniques to upregulate activity may help an individual gain the ability to modulate their gait speeds.

## Figures and Tables

**Table 1:** Summary of participant details.

	*N* = 20
Age [mean (SD)]	64 (7.6) years
Sex (female/male)	7/13
Chronicity [mean (SD)]	82 (67.4) months
Lesion depth (cortical/subcortical/mixed)	0/17/3
Lesion side (left/right)	7/13
FM-LE (34 max)	27 (4.9)
MoCA (30 max)	26 (2.6)
Gait aids (none/walking stick(s)/4-point cane/4 wheeled walker)	12/6/1/1
Normal gait speed [mean (SD)]	0.83 (0.346) m/s Range: 0.14–1.39 m/s
Slow gait speed [mean (SD)]	0.64 (0.34) m/s ^*∗*^ Range: 0.10–1.32 m/s *t*(19) = 3.05, *p*=0.007, CI = 0.06*–*0.31
Fast gait speed [mean (SD)]	1.08 (0.45) m/s ^*∗*^ Range: 0.14–1.88 m/s *t*(19) = −7.65, *p* < 0.001, CI = −0.30 to −0.17

*Note*:  ^*∗*^Significantly different than the *NORM* condition. FM-LE = Fugl-Meyer lower extremity; MoCA = Montreal Cognitive Assessment; CI = 95% confidence interval.

**Table 2:** Linear mixed-effects models results.

ROI	Predictors	Estimates	Confidence interval	*p*	ICC	*N*_subj_	Observations	Marginal *R*^2^/conditional *R*^2^
(a). Aim 1: laterality index ∼ condition + (1|participant)								

PFC	(Intercept)	0.057449	−0.167247–0.282145	0.610	0.74	20	60	0.011/0.739
	Condition (*FAST*)	−0.114453	−0.277834–0.0489273	0.166				
	Condition (*SLOW*)	−0.004908	−0.168288–0.158473	0.952				

PMC	(Intercept)	0.053142	−0.191337–0.297621	0.665	0.73	20	60	0.020/0.737
	Condition (*FAST*)	−0.149032	−0.328234–0.030171	0.101				
	Condition (*SLOW*)	0.027203	−0.152000–0.206405	0.762				

SMC	(Intercept)	0.164832	−0.115489–0.445153	0.243	0.72	18	54	0.001/0.720
	Condition (*FAST*)	−0.054382	−0.264360–0.155596	0.605				
	Condition (*SLOW*)	−0.022304	−0.232282–0.187674	0.832				

PPC	(Intercept)	−0.002626	−0.231353–0.226100	0.982	0.51	19	57	0.003/0.516
	Condition (*FAST*)	−0.007294	−0.232649–0.218060	0.948				
	Condition (*SLOW*)	0.049297	−0.176058–0.274652	0.663				

(b). Aim 2: HbO ∼ condition + Hemisphere + (1|participant) and HbO ∼ condition + (1|participant)								

PFC	(Intercept)	0.091497	−0.006430–0.189424	0.067	0.34	20	801	0.011/0.345
	Condition (*FAST*)	0.033987	−0.014290–0.082264	0.167				
	Condition (*SLOW*)	0.000406	−0.047871–0.048683	0.987				
	Hemisphere (ipsi)	0.067867	0.027958–0.107776	*0.001* ^*∗*^				

PMC	(Intercept)	0.048776	−0.059401–0.156953	0.376	0.38	20	636	0.007/0.383
	Condition (*FAST*)	0.069844	0.013942–0.125746	*0.014*				
	Condition (*SLOW*)	0.060211	0.004309–0.116113	*0.035*				

SMC	Intercept)	0.089338	−0.018620–0.197295	0.105	0.37	20	441	0.003/0.375
	Condition (*FAST*)	0.044168	−0.021419–0.109755	0.186				
	Condition (*SLOW*)	0.004477	−0.061110–0.070063	0.893				

PPC	(Intercept)	0.001145	−0.111003–0.113293	0.984	0.32	19	471	0.016/0.332
	Condition (*FAST*)	0.119288	0.048082–0.190495	*0.001* ^*∗*^				
	Condition (*SLOW*)	0.077668	0.006462–0.148875	*0.033*				

*Note*: Predictors indicate the fixed effects levels within the variables in the model. Reference level was the *NORM* condition and the contralesional hemisphere, when applicable. Estimates indicate the difference between the reference level and the predictor level. Italic *p*-values indicate a significant difference at alpha of 0.05.  ^*∗*^Indicates significant differences with *p* ≤ 0.0125 (0.05/4: Bonferroni correction for four models). PFC = prefrontal cortex; PMC = premotor cortex; SMC = sensorimotor cortex; PPC = posterior parietal cortex; ipsi = ipsilesional hemisphere.

**Table 3:** Pearson's correlation results for Aim 3 with comparisons to between brain activation changes, gait speed modulation, and impairment.

Region of interest		Gait speed modulation				Impairment (Fugl-Meyer lower extremity)			
		*NORM* minus *SLOW*		*FAST* minus *NORM*		*NORM* minus *SLOW*		*FAST* minus *NORM*	
		*r*	*p*	*r*	*p*	*r*	*p*	*r*	*p*
Contralesional hemisphere	PFC	−0.222	0.360	0.402	0.079	0.009	0.969	0.323	0.165
	PMC	−0.326	0.173	−0.183	0.439	−0.006	0.981	−0.155	0.513
	SMC	−0.234	0.350	0.237	0.330	0.388	0.101	0.375	0.114
	PPC	−0.295	0.234	0.353	0.138	0.208	0.394	0.235	0.332

Ipsilesional hemisphere	PFC	−0.282	0.241	0.598	*0.005* ^*∗*^	0.172	0.469	0.503	*0.024*
	PMC	−0.310	0.197	0.204	0.387	−0.118	0.620	0.250	0.287
	SMC	−0.104	0.682	0.201	0.410	−0.025	0.919	0.249	0.303
	PPC	−0.382	0.117	0.137	0.576	0.186	0.446	0.242	0.318

*Note*: Change in brain activation and gait speed modulations were calculated as a change from the *SLOW* to *NORM* condition and change from the *NORM* to *FAST* condition. Italic *p*-values indicate significant relationships with an alpha of 0.05.  ^*∗*^Indicates significant relationships after Benjamini–Hochberg correction for multiple comparisons (using a false discovery rate of 5%). PFC = prefrontal cortex; PMC = premotor cortex; SMC = sensorimotor cortex; PPC = posterior parietal cortex.

## Data Availability

The imaging data in Excel format used to support the findings of this study have been deposited into Janice Eng's Dataverse on the Borealis Canadian Dataverse Repository.

## Additional Points

*Limitations*. Our previous systematic review consolidating investigations of brain activation during walking in the stroke population [55] found 22 studies with an average of 13 participants in the studies. Although our sample size exceeded this average, we acknowledge that we were not powered to detect some smaller effects and our results are limited to our small sample size. In addition, a lack of a healthy older-adult group limits some of our interpretations of whether the results observed are attributed to the effect of stroke or aging. However, our hemisphere-dependent findings suggest that our results are not exclusively a product of aging and some stroke-specific compensations are needed when walking at different speeds.

We acknowledge that walking at different speeds, particularly for the *FAST* condition, may have introduced more motion artifacts compared to the slower speeds [56]. Unfortunately, we are not able to calculate the number of motion artifacts within each condition, however, upon visual inspection of the raw data motion artifacts were not more abundant during the *FAST* condition for the majority of participants. The exact speed of walking and the change in walking speed was not uniform across all participants. This was done intentionally to determine if the participants' ability to modulate their walking speed was related to their brain activity. As a result of the different walking speeds, we introduced more variability and may have reduced the ability to detect brain activations. Future studies should investigate if brain activity changes are related to specific increases or decreases in gait speed.

## Supplementary Materials

Supplementary Materials Table S1: individual participant demographics and performance details. Table S2: channel numbers associated with regions of interest and excluded channel due to lesion or preprocessing. Table S3: number of channels excluded by region. Table S4: results from Aim 1 and 2 for deoxyhemoglobin (HbR) results. Table S5: Pearson's correlation results for Aim 3 with comparisons to between brain activation changes, gait speed modulation, and impairment.
