# Supplementary material for: Premotor and Posterior Parietal Cortex Activity is Increased for Slow, as well as Fast Walking Poststroke: An fNIRS Study
Source: Neural Plast. 2023 Oct 13;2023:2403175. doi: 10.1155/2023/2403175 (PMC10589070; doi:10.1155/2023/2403175)
Supplement: Supplementary Materials — Table S1: individual participant demographics and performance details. Table S2: channel numbers associated with regions of interest and excluded channel due to lesion or preprocessing. Table S3: number of channels excluded by region. Table S4: results from Aim 1 and 2 for deoxyhemoglobin (HbR) results. Table S5: Pearson's correlation results for Aim 3 with comparisons to between brain activation changes, gait speed modulation, and impairment. [file 2403175.f1.docx]

Table 1. Individual participant demographics and performance details

| Participant ID | Age | Sex | Stroke chronicity (months) | Lesion side and depth | FMLE (/34) | Normal-pace gait speed (m/s) | Slow gait speed (m/s) | Fast gait speed (m/s) |
| --- | --- | --- | --- | --- | --- | --- | --- | --- |
| S01 | 63 | M | 21 | R subcortical | 31 | 1.19 | 1 | 1.35 |
| S02 | 58 | M | 6 | R subcortical | 27 | 0.87 | 0.9 | 1.05 |
| S03 | 67 | M | 46 | R subcortical | 29 | 0.8 | 0.61 | 1.19 |
| S04 | 61 | F | 15 | L mixed | 29 | 0.66 | 0.64 | 0.89 |
| S05 | 58 | F | 21 | R mixed | 19 | 0.14 | 0.1 | 0.14 |
| S06 | 49 | F | 55 | R subcortical | 30 | 0.72 | 0.62 | 0.86 |
| S07 | 50 | F | 13 | R subcortical | 33 | 1.36 | 0.13 | 1.88 |
| S08 | 67 | M | 115 | L subcortical | 28 | 1.39 | 1.32 | 1.6 |
| S09 | 62 | F | 230 | L subcortical | 26 | 0.57 | 0.51 | 0.82 |
| S10 | 54 | M | 12 | R subcortical | 20 | 0.78 | 0.32 | 1.24 |
| S11 | 73 | M | 162 | R subcortical | 32 | 1.11 | 0.96 | 1.3 |
| S12 | 67 | M | 67 | R subcortical | 27 | 0.95 | 0.84 | 1.24 |
| S13 | 72 | M | 144 | R mixed | 24 | 0.54 | 0.51 | 0.67 |
| S14 | 63 | M | 38 | R subcortical | 29 | 0.9 | 0.74 | 1.2 |
| S15 | 69 | F | 159 | L subcortical | 18 | 0.18 | 0.15 | 0.21 |
| S16 | 72 | M | 126 | R unknown | 19 | 0.51 | 0.38 | 0.66 |
| S17 | 69 | M | 50 | L subcortical | 29 | 0.85 | 0.5 | 0.95 |
| S18 | 74 | F | 128 | R subcortical | 31 | 0.71 | 0.59 | 0.98 |
| S19 | 71 | M | 52 | L subcortical | 24 | 1.19 | 1.15 | 1.6 |
| S20 | 71 | M | 179 | L unknown | 34 | 1.16 | 0.9 | 1.5 |

Table 2. Channel numbers associated with regions of interests and excluded channels due to lesion or preprocessing

| Participant | Prefrontal cortex (PFC) | | Premotor cortex (PMC) | | Sensorimotor cortex (SMC) | | Posterior parietal cortex (PPC) | |  |
| --- | --- | --- | --- | --- | --- | --- | --- | --- | --- |
|  | Ipsilesional | Contralesional | Ipsilesional | Contralesional | Ipsilesional | Contralesional | Ipsilesional | Contralesional | |
| S01 | **8 channels:**  7; 8; 9; 10; 11; 16; 21; 22 | **7 channels:**  1; 2; 4; 5; 6; 13; ~~14~~ | **7 channels:** 18; 19; 23; 30; 31; 32; 40 | **7 channels:**  15; 17; 24; 25; 26; 27; 28 | **4 channels:** 33; 35; 45; 46 | **3 channels:**  41; 36; 37 | **6 channels:**  42; 43; ~~47;~~ 52; 53; 54 | **4 channels:**  38; 49; 50; 51 | |
| S02 | **8 channels:**  7; 8; 9; 10; 11; 16; ~~21;~~ 22 | **8 channels:**  1; 2; 4; 5; 6; 13; 14; 17 | **6 channels:** 18; ~~23; 30;~~ 31; 32; 33 | **7 channels:**  15; 19; ~~25;~~ ~~26; 27; 28; 40~~ | **3 channels:** 35; ~~45;~~ 46 | **4 channels:** ~~24; 41;~~ ~~36; 37~~ | **6 channels:**  42; 43; 47; ~~52; 53; 54~~ | **4 channels:**  38; 49; 50; 51 | |
| S03 | **8 channels:**  7; 8; 9; 10; 11; 16; 21; 22 | **7 channels:**  1; 2; 4; 5; 6; 13; 17 | **6 channels:** 18; 23; 30; 31; 32; 35 | **8 channels:**  14; 15; 19; 25; 26; 27; 28; 40 | **3 channels:** 33; 45; 46 | **4 channels:** 24; 36; 37; 41 | **5 channels:**  42; 47; 52; 53; 54 | **5 channels:**  38; 43; 49; 50; 51 | |
| S04 | **6 channels:**  1; 2; 4; 14; 13; 6 | **8 channels:**  5; 7; 8; 9; 10; 11; 16; 21; 22 | **5 channels:** 15; 17; 25; 26; 27 | **6 channels:**  18; 19; 23; 30; 31; 32 | **6 channels:** ~~28;~~ 36; 40; 24; 37; 41 | **3 channels:** 33; 35; 45 | **5 channels:**  38; 43; 49; ~~50;~~ 51 | **6 channels:**  42; 46; ~~47;~~ 52; 53; ~~54~~ | |
| S05 | **7 channels:**  7; ~~8; 10; 11; 16;~~ ~~21; 22~~ | **7 channels:**  ~~1; 2;~~ 4; 5; ~~6;~~ ~~13;~~ 14; | **7 channels:**  ~~9;~~ 18; ~~19; 23; 30; 31; 32~~ | **6 channels:**  17; 18; 15; 25; 26; 27; 40 | **4 channels:** ~~33; 35; 45; 46~~ | **4 channels:**  24; 28; 37; 41 | **5 channels:**  ~~42; 52; 47; 53; 54~~ | **6 channels:**  36; ~~38;~~ 43; 49; ~~50; 51~~ | |
| S06 | **8 channels:**  7; 8; 9; 10; 11; ~~16;~~ 21; 22 | **5 channels:**  1; 2; 4; 5; 13 | **7 channels:** ~~18;~~ 19; 23; ~~30;~~ 31; ~~32;~~ 35 | **10 channels:**  ~~6;~~ 14; 15; 17; 24; ~~25; 26;~~ 27; 28; 40 | **3 channels:** 33; 42; 45 | **2 channels:** 36; 37 | **5 channels:**  46; 47; ~~52; 53;~~ 54 | **6 channels:**  38; 41; 43; 49; ~~50; 51~~ | |
| S07 | **7 channels:**  7; 8; 10; 11; 16; 21; 22 | **6 channels:**  ~~1;~~ 2; 4; 5; ~~6;~~ 13 | **7 channels:**  ~~9;~~ 18; 19; 23; ~~30; 31;~~ 32 | **7 channels:**  ~~14;~~ 15; 17; ~~25;~~ 26; ~~27;~~ 40 | **4 channels:** 33; 35; 45; 46 | **5 channels:** 24; 28; 36; 37; 41 | **5 channels:**  ~~42;~~ 47; ~~52; 53;~~ 54 | **5 channels:**  38; 43; 49; 50; 51 | |
| S08 | **6 channels:**  1; 2; 4; 5; 6; 13 | **7 channels:**  7; 8; 10; 11; 16; 21; 22 | **7 channels:** 14; 15; 17; 25; 26; 27; 40 | **7 channels:**  9; 18; 19; 23; 30; 31; 32 | **4 channels:** 24; 28; 36; 37 | **5 channels:** 33; 35; 42; 45; 46 | **6 channels:**  38; 41; 43; 49; 50; 51 | **4 channels:**  47; 52; 53; 54 | |
| S09 | **6 channels:**  1; 2; 4; 5; ~~6;~~ 13 | **8 channels:**  7; 8; 9; ~~10;~~ 11; ~~16;~~ 21; 22 | **7 channels:** 14; 15; ~~17;~~ 25; 26; ~~27; 40~~ | **6 channels:**  18; ~~19;~~ 23; ~~30;~~ 31; 32 | **4 channels:** 24; 28; 36; 37 | **5 channels:** 33; 35; 42; 45; 46 | **5 channels:**  38; ~~41;~~ 49; 50; ~~51~~ | **5 channels:**  ~~43;~~ 47; 52; ~~53; 54~~ | |
| S10 | **8 channels:**  7; 8; 9; 10; 11; 16; 21; 22 | **7 channels:**  1; 2; 4; 5; 6; 13; 17 | **6 channels:** 18; 19; 23; 30; 31; 32 | **6 channels:**  14; 15; 25; 26; 27; 28 | **4 channels:** 33; 35; 45; 46 | **5 channels:** 24; 36; 37; 40; 41 | **5 channels:**  42; 47; 52; 53; 54 | **5 channels:**  38; 43; 49; 50; 51 | |
| S11 | **7 channels:**  7; 8; 10; 11; 16; 21; 22 | **8 channels:**  1; 2; 4; 5; 6; 13; 14; 17 | **8 channels:**  9; 18; ~~19;~~ 23; ~~30; 31;~~ 32; ~~33~~ | **7 channels:**  15; 24; 25; 26; 27; 28; ~~40~~ | **4 channels:** 35; 45; 46; 52 | **3 channels:** 36; 37; 41 | **3 channels:**  47; ~~53;~~ 54 | **5 channels:**  38; 43; 49; 50; 51 | |
| S12 | **8 channels:**  7; 8; 9; 10; 11; 16; 21; 22 | **6 channels:**  1; 2; 4; 5; 6; 13 | **7 channels:** 18; 19; 23; 30; 31; 32; 33 | **8 channels:**  14; 15; 17; 25; 26; 27; 28; 40 | **4 channels:** 35; 42; 45; 46 | **4 channels:** 24; 36; 37; 41 | **5 channels:**  43; 47; 52; 53; 54 | **4 channels:**  38; 49; 50; 51 | |
| S13 | **7 channels:**  7; 8; 9; 10; 11; 21; 22 | **8 channels:**  1; 2; 4; 5; ~~6;~~ ~~13;~~ 16; 17 | **5 channels:** 18; 23; ~~30;~~ 31; 32 | **7 channels:**  14; 15; ~~19;~~ ~~25;~~ 26; 27; ~~40~~ | **4 channels:** 33; 35; 45; 46 | **5 channels:** 24; 28; 36; 37; 41 | **6 channels:**  42; 43; 47; ~~52;~~ 53; 54 | **4 channels:**  38; ~~49;~~ 50; 51 | |
| S14 | **8 channels:**  7; 8; 9; 10; 11; 16; 21; 22 | **8 channels:**  1; 2; 4; 5; 6; 13; 14; ~~17~~ | **6 channels:** ~~18;~~ 19; 23; 30; 31; 32 | **5 channels:**  15; ~~25;~~ 26; 27; 40 | **4 channels:** 33; 35; 42; 45 | **5 channels:** 24; 28; 41; 36; 37 | **5 channels:**  43; 47; 52; 53; 54 | **4 channels:**  38; 49; 50; 51 | |
| S15 | **8 channels:**  1; 2; 4; 5; 6; 13; 14; 17 | **8 channels:**  7; 8; 9; 10; 11; 16; 21; 22 | **8 channels:** 15; 19; 24; 25; 26; 27; 28; 40 | **5 channels:**  18; 23; 30; 31; 32 | **3 channels:** 36; 37; 41 | **5 channels:** 33; 35; 42; 45; 46 | **5 channels:**  38; 43; 49; 50; 51 | **4 channels:**  47; 52; 53; 54 | |
| S16 | **9 channels:**  5; 7; 8; 9; 10; ~~11;~~ 16; 21; 22 | **5 channels:**  1; 2; 4; 6; 13 | **6 channels:** 18; 19; 23; 30; 31; 32 | **6 channels:**  14; 15; 17; 25; 26; 27 | **3 channels:** 33; 35; 45 | **6 channels:**  24; 28; 36; 37; 40; 41 | **6 channels:**  42; 46; 47; 52; 53; 54 | **5 channels:**  38; 43; 49; 50; 51 | |
| S17 | **8 channels:**  1; 2; 4; ~~5;~~ 6; 13; 14; 17 | **7 channels:**  7; 8; 9; 10; 11; 16; 21; 22 | **6 channels:** 15; 24; 25; 26; 27; 28; 40 | **6 channels:**  18; 19; 23; 30; 31; 32 | **3 channels:** 36; 37; 41 | **2 channels:**  33; 35 | **5 channels:**  38; 43; 49; 50; 51 | **6 channels:**  42; 46; 47; 52; 53; 54 | |
| S18 | **10 channels:**  5; 7; 8; 9; 10; 11; ~~16; 18;~~ 21; 22 | **5 channels:**  1; ~~2;~~ 4; ~~6;~~ 13 | **5 channels:** 23; ~~30; 31;~~ 32; ~~33~~ | **9 channels:**  14; 15; 17; ~~19; 25; 26; 27; 28;~~ 40 | **4 channels:** 35; 45; 42; 46 | **4 channels:**  24; 36; 37; 41 | **4 channels:**  ~~47; 52; 53; 54~~ | **5 channels:**  ~~38; 43;~~ ~~49; 50; 51~~ | |
| S19 | **7 channels:**  1; 2; 4; 6; 13; 14; 17 | **9 channels:**  5;~~6;~~ 7; 8; 9;10; 11; 16; 21; 22 | **5 channels:** 15; 25; 26; 27; 28 | **6 channels:**  18; 19; 23; 30; 31; 32 | **4 channels:** 24; 36; 37; 41 | **6 channels:**  33; 35; 40; 42; 45; 46 | **4 channels:**  38; 49; 50; 51 | **5 channels:**  43; 47; 52; 53; 54 | |
| S20 | **8 channels:**  1; 2; 4; 5; 6; 13; 14; 17 | **8 channels:**  7; 8; 9; 10; 11; 16; 21; 22 | **5 channels:** 15; 25; 27; 28; 40 | **5 channels:**  18; 19; 23; 30; 31 | **4 channels:** 24; 26; 36; 37 | **5 channels:**  32; 33; 35; 45; 46 | **6 channels:**  38; 41; 43; 49; 50; 51 | **5 channels:**  42; 47; 52; 53; 54 | |

Highlighted cells indicate regions with excluded channels. Numbers with a strike through indicate channel numbers that were excluded after pre-processing. Numbers with a strike through and underline indicate channel numbers recorded over lesion area and thus excluded.

**Table 3. Number of channels excluded by region**

|  | **Ipsilesional** | **Contralesional** |
| --- | --- | --- |
| **PFC** | 7 | 15 |
| **PMC** | 21 | 24 |
| **SMC** | 6 | 8 |
| **PPC** | 18 | 17 |

Table 4. Linear mixed-effects models results

| 1. **Aim 1: laterality index~ Condition + (1\|Participant)** | | | | | | | | |
| --- | --- | --- | --- | --- | --- | --- | --- | --- |
| ROI | Predictors | Estimates | Confidence Interval | p | ICC | N_subj_ | Observations | Marginal R^2^ / Conditional R^2^ |
| PFC | (Intercept) | 0.044141 | -0.262515 – 0.350798 | 0.774 | 0.52 | 20 | 60 | 0.000 / 0.518 |
|  | Condition [*Fast*] | 0.012029 | -0.289004 – 0.313062 | 0.936 |  |  |  |  |
|  | Condition [*Slow*] | -0.024066 | -0.325099 – 0.276966 | 0.873 |  |  |  |  |
| PMC | (Intercept) | -0.302234 | -0.613237 – 0.008770 | 0.057 | 0.60 | 20 | 60 | 0.005 / 0.602 |
|  | Condition [*Fast*] | 0.113262 | -0.164902 – 0.391426 | 0.418 |  |  |  |  |
|  | Condition [*Slow*] | 0.100543 | -0.177621 – 0.378708 | 0.472 |  |  |  |  |
| SMC | (Intercept) | -0.269656 | -0.601044 – 0.061733 | 0.109 | 0.74 | 19 | 57 | 0.059 / 0.752 |
|  | Condition [*Fast*] | 0.368781 | 0.128314 – 0.609248 | **0.003*** |  |  |  |  |
|  | Condition [*Slow*] | 0.389549 | 0.149082 – 0.630016 | **0.002*** |  |  |  |  |
| PPC | (Intercept) | 0.148336 | -0.198066 – 0.494739 | 0.394 | 0.75 | 18 | 54 | 0.002 / 0.754 |
|  | Condition [*Fast*] | 0.045761 | -0.197516 – 0.289039 | 0.707 |  |  |  |  |
|  | Condition [*Slow*] | -0.037784 | -0.281062 – 0.205494 | 0.756 |  |  |  |  |
| 1. **Aim 2: HbO ~ Condition + Hemisphere + (1\|Participant)** *and* **HbO ~ Condition + (1\|Participant)** | | | | | | | | |
| ROI | Predictors | Estimates | Confidence Interval | p | ICC | N_subj_ | Observations | Marginal R^2^ / Conditional R^2^ |
| PFC | (Intercept) | -0.014562 | -0.041454 – 0.012330 | 0.288 | 0.10 | 20 | 801 | 0.006 / 0.110 |
|  | Condition [*Fast*] | 0.006323 | -0.015717 – 0.028364 | 0.573 |  |  |  |  |
|  | Condition [*Slow*] | -0.001255 | -0.023296 – 0.020785 | 0.911 |  |  |  |  |
|  | Hemisphere [ipsi] | 0.020013 | 0.001822 – 0.038204 | **0.031** |  |  |  |  |
| PMC | (Intercept) | -0.026327 | -0.063290 – 0.010635 | 0.162 | 0.15 | 20 | 636 | 0.009 / 0.158 |
|  | Condition [*Fast*] | -0.014782 | -0.044102 – 0.014538 | 0.323 |  |  |  |  |
|  | Condition [*Slow*] | 0.003157 | -0.026163 – 0.032477 | 0.833 |  |  |  |  |
|  | Hemisphere [ipsi] | -0.028054 | -0.052351 – -0.003758 | **0.024** |  |  |  |  |
| SMC | (Intercept) | -0.027558 | -0.058991 – 0.003874 | 0.086 | 0.37 | 20 | 441 | 0.003 / 0.375 |
|  | Condition [*Fast*] | -0.003979 | -0.034426 – 0.026468 | 0.797 |  |  |  |  |
|  | Condition [*Slow*] | -0.002040 | -0.032487 – 0.028407 | 0.895 |  |  |  |  |
|  | Hemisphere [ipsi] | 0.029950 | 0.004393 – 0.055507 | **0.022** |  |  |  |  |
| PPC | (Intercept) | -0.039877 | -0.078339 – -0.001414 | **0.042** | 0.16 | 19 | 471 | 0.001 / 0.157 |
|  | Condition [*Fast*] | 0.005941 | -0.028012 – 0.039894 | 0.731 |  |  |  |  |
|  | Condition [*Slow*] | 0.011487 | -0.022467 – 0.045440 | 0.507 |  |  |  |  |

Predictors indicate the fixed effects levels within the variables in the model. Reference level was the *NORM* Condition and the contralesional hemisphere, when applicable. Estimates indicate the difference between the reference level and the predictor level. Bolded values indicate a significant difference at alpha of 0.05. * indicates significant differences with p≤0.0125 (0.05 /4: Bonferroni correction for four models). PFC=prefrontal cortex, PMC=premotor cortex, SMC=sensorimotor cortex, PPC=posterior parietal cortex, ipsi=ipsilesional hemisphere.

Table 5. Pearson’s correlation results for Aim 3 with comparisons to between brain activation changes, gait speed modulation, and impairment

| Region of Interest | | *Gait speed modulation* | | | | *Impairment (Fugl-Meyer lower extremity)* | | | |
| --- | --- | --- | --- | --- | --- | --- | --- | --- | --- |
|  |  | *NORM* minus *SLOW* | | *FAST* minus *NORM* | | *NORM* minus *SLOW* | | *FAST* minus *NORM* | |
|  |  | *r* | *p* | *r* | *p* | *r* | *p* | *r* | *p* |
| Contralesional hemisphere | PFC | -0.121 | 0.622 | -0.086 | 0.720 | 0.174 | 0.463 | 0.174 | 0.463 |
|  | PMC | -0.063 | 0.799 | -0.104 | 0.664 | -0.109 | 0.648 | -0.109 | 0.648 |
|  | SMC | 0.016 | 0.950 | 0.225 | 0.353 | -0.066 | 0.788 | -0.066 | 0.788 |
|  | PPC | -0.154 | 0.542 | -0.225 | 0.353 | -0.179 | 0.462 | -0.179 | 0.462 |
| Ipsilesional hemisphere | PFC | -0.103 | 0.675 | -0.405 | 0.076 | -0.230 | 0.328 | -0.230 | 0.328 |
|  | PMC | -0.173 | 0.478 | -0.412 | 0.071 | -0.206 | 0.384 | -0.206 | 0.384 |
|  | SMC | -0.015 | 0.953 | -0.003 | 0.990 | 0.007 | 0.978 | 0.007 | 0.978 |
|  | PPC | -0.348 | 0.156 | -0.352 | 0.139 | -0.301 | 0.210 | -0.301 | 0.210 |

Change in brain activation and gait speed modulations were calculated as a change from the *SLOW* to *NORM* condition and change from the *NORM* to *FAST* condition. Bolded p-values indicate significant relationships with an alpha of 0.05. *Indicates significant relationships after Benjamini-Hochberg correction for multiple comparisons (using a false discovery rate of 5%). PFC=prefrontal cortex, PMC=premotor cortex, SMC=sensorimotor cortex, PPC=posterior parietal cortex.
